# Publishing Open, Reproducible Research With Undergraduates

**DOI:** 10.3389/fpsyg.2019.00564

**Published:** 2019-03-20

**Authors:** Julia F. Strand, Violet A. Brown

**Affiliations:** ^1^Department of Psychology, Carleton College, Northfield, MN, United States; ^2^Department of Psychological & Brain Sciences, Washington University in St. Louis, St. Louis, MO, United States

**Keywords:** open science, undergraduate, training, pre-registration, replicability

In response to growing concern in psychology and other sciences about low rates of replicability of published findings (Open Science Collaboration, 2015), there has been a movement toward conducting open and transparent research (see Chambers, 2017). This has led to changes in statistical reporting guidelines in journals (Appelbaum et al., 2018), new professional societies (e.g., Society for the Improvement of Psychological Science), frameworks for posting materials, data, code, and manuscripts (e.g., Open Science Framework, PsyArXiv), initiatives for sharing data and collaborating (e.g., Psych Science Accelerator, Study Swap), and educational resources for teaching through replication (e.g., Collaborative Replications and Education Project). This “credibility revolution” (Vazire, 2018) provides many opportunities for researchers. However, given the recency of the changes and the rapid pace of advancements (see Houtkoop et al., 2018), it may be overwhelming for faculty to know whether and how to begin incorporating open science practices into research with undergraduates.

In this paper, we will not attempt to catalog the entirety of the open science movement (see recommended resources below for more information), but will instead highlight why adopting open science practices may be particularly beneficial to conducting and publishing research with undergraduates. The first author is a faculty member at Carleton College (a small, undergraduate-only liberal arts college) and the second is a former undergraduate research assistant (URA) and lab manager in Dr. Strand's lab, now pursuing a PhD at Washington University in St. Louis. We argue that open science practices have tremendous benefits for undergraduate students, both in creating publishable results and in preparing students to be critical consumers of science.

## Reading

A simple way to introduce open science practices is to ask URAs to read papers related to the replication crisis, as this may be novel content even for those who have taken a research methods class. When students join the lab, we typically spend one lab meeting discussing *False Positive Psychology* (Simmons et al., 2011, see also Simmons et al., 2018), an engaging introduction to *researcher degrees of freedom*—the choices made during the research process that enable researchers to “publish ‘statistically significant' evidence consistent with any hypothesis” (Simmons et al., 2011). Articles like this, or Chris Chambers' book *The Seven Deadly Sins of Psychology* (Chambers, 2017), are more accessible than empirical articles to inexperienced lab students. These readings allow URAs to engage with the material and contribute to group discussions more quickly than they typically can for research content, which requires greater familiarity with the literature and discipline-specific conventions. These readings can inform students about questionable research practices (John et al., 2012) that increase the likelihood of Type I error, such as Hypothesizing After the Results are Known (HARKing; Kerr, 1998) or *p*-hacking (conducting multiple analyses and only reporting those that render statistically significant results). Once students are familiar with these topics, we point out places in our own research process where bias could enter (e.g., “How should we decide what counts as an outlier in a reaction time task? When should we make those decisions?”), and discuss how to combat these biases.

## Writing and Pre-registering

We begin new projects by collaboratively writing a manuscript-style proposal containing detailed introduction, methods, and analysis sections. Writing the paper before we conduct the study means that incoming URAs have a reference document that they can read and review independently prior to group discussion. We have found that this is far more effective at helping new students master the content than referring them to related published papers and giving brief verbal descriptions of the new project. As a result, students are more able and willing to contribute early on, and therefore more quickly feel like members of the lab community. Further, given that one role for new URAs is often to collect data for ongoing experiments, the methods section in the project proposal can serve as a less daunting avenue for asking questions than the theory-driven introduction section.

As an assignment for lab meeting, we ask new URAs to write about the consequences of certain methodological decisions (e.g., between- vs. within-subjects design or blocked vs. intermixed trials), and have returning students contribute to writing the introduction section of the research proposal. We have found that this exercise not only benefits students, but also helps us notice potential methodological shortcomings ahead of time. Armed with a more thorough understanding of the literature and methodological considerations, students have the knowledge and experience to play a more substantial role in the next project, and consequently become authors on published papers earlier in their academic careers.

A clear benefit to writing project proposals ahead of time is that it relieves the burden of writing the introduction and methods sections later, when the theoretical background is no longer fresh in mind. This is work that must be completed eventually if the project is going to be submitted for publication, and can make the writing process less daunting later on, particularly for URAs with little experience. Indeed, this process can cut down on the number of datasets waiting to be written up because the amount of work that is required to turn the project proposal into a manuscript is minimal. This rapid publication rate has proved extremely beneficial for undergraduates, as student co-authors have the opportunity to see the submission and review process from start to finish. Thus, writing a project proposal with URAs helps them become involved early in their research career, which increases the number of projects to which they can make substantial contributions, and encourages them to publish findings that otherwise may not have made it into the scientific record.

Once we have finalized the proposal, we pre-register the project on the Open Science Framework (OSF). Pre-registration involves creating a timestamped, uneditable document containing hypotheses (or research questions) and analysis plans (Wagenmakers et al., 2012; Lindsay et al., 2016; Nosek et al., 2018 for more information). It is important to note that a pre-registration is “a plan, not a prison” (DeHaven, 2017); if you realize you need to deviate from your pre-registered plan, you simply explain in the manuscript how and why you did so. Thus, pre-registration makes clear which analyses were confirmatory (pre-registered) versus exploratory (not pre-registered). An eventual manuscript can then link to the pre-registration document to demonstrate that the experiment reported is consistent with the experiment planned (e.g., all conditions are reported, data exclusions are justified, analyses were planned, etc.), and therefore helps combat HARKing and *p*-hacking.

A benefit of pre-registration is that in our experience, it has made it easier to publish interesting and informative null results. Two of our lab's recent publications included unexpected null findings—in both cases we had theory-driven hypotheses about directional effects, so the null effects make important theoretical contributions to the field. Data like these are liable to languish in the file drawer (e.g., see Rosenthal, 1979; Chambers, 2017), but given that most of the writing was already done, the work needed to finish the papers was relatively light. Reviewers have been overwhelmingly positive about pre-registration, leading us to believe that they are more accepting of theoretically interesting null results when the hypotheses are pre-registered (note that pre-registration is a relatively recent development, so there is not yet data on whether it systematically affects the likelihood of publication).

## Sharing Data and Materials

At the time of manuscript submission, we make all of our data, code, and stimuli publicly available on the OSF—a practice that reviewers consistently praise. Not only does transparency benefit the research community at large by facilitating re-use of stimuli, independent examination of results, the potential for re-analysis or meta-analysis, and examples of how to conduct statistical analyses (see Klein et al., 2018), but this practice can also benefit the researchers themselves. In one recent paper of ours, a reviewer recommend a change to how we presented data in a figure. Instead of simply describing the change, they accessed the code, edited it to make the change, and included the updated code and altered figure with their review. In addition, open research is associated with more citations, increased media coverage, and improved funding opportunities (McKiernan et al., 2016).

Knowing that others can see our code means writing and commenting much more carefully than we might do for just ourselves. To ensure the transparency of our analyses, we use our own R (R Core Team, 2016) scripts as reading assignments for lab meetings. Given that we do not require that incoming URAs have statistical backgrounds, the code must be commented carefully enough that a naïve reader can interpret it. The biggest benefit we have found to writing code this way is that the script becomes a valuable resource for future students. Not only can these scripts expose students without statistical background to coding in R, but they can also serve as excellent templates for conducting future analyses, thereby streamlining data analysis for subsequent publications. Instilling good habits by writing clean, commented code also helps URAs build a strong foundation for graduate school, where learning statistics and R can be daunting if they have never been exposed to them.

## Connecting With the Open Science Community

Finally, we have found that transitioning to open science practices has been helped by connecting with others. In person, this has involved attending the Society for the Improvement of Psychological Science meeting and related meet-ups at conferences. Connecting with the open science community digitally has also proved valuable through blogs, podcasts, and Twitter. There is active and spirited discussion about open science on Twitter, and we have found it to be very effective for staying up to date with issues and advancements, discovering new papers, getting rapid answers to questions, and networking. Indeed, one of the studies currently underway in our lab is a collaboration with a colleague we initially connected with via Twitter. This joint venture is a project neither lab is likely to have conducted alone, so this experience can serve as an example to URAs of the potential professional benefits of digital networking.

Though it might seem unprofessional to include social media as a recommendation, Twitter is currently the primary platform on which open science researchers communicate. Research practices are changing quickly, and though publications about research transparency are certainly valuable, they may be more limited in scope, speed, and breadth of views than the conversations that occur on social media. An additional benefit to URAs of becoming involved in the open science community online is that it becomes easier to approach senior researchers in person (e.g., at conferences) when they are familiar with each other online. Therefore, making digital contact can facilitate students forming professional connections that may benefit future careers.

## Conclusions

Although there are benefits to introducing open science practices at any stage, it may be particularly fruitful for undergraduates. A given URA is less likely to pursue a career in their lab's research area than a graduate student is, so broad training in open science and meta-science may help provide more generalizable knowledge than learning only area-specific techniques would. URAs may also be particularly receptive to these approaches because they are likely to tend to think that “calling your shots” and being transparent is how science should work. That is, being naïve scientists makes them the perfect audience. Finally, given the disciplinary shift toward using open science practices (e.g., Kidwell et al., 2016; Nosek et al., 2018), early experience is likely to benefit the careers of students going into research.

Importantly, the practices described here can be incorporated incrementally and piecemeal into existing research programs. We began adding these practices to our lab in roughly the order that we describe them, and have found considerable benefits to our lab and our students that far outweigh any costs of adopting new practices.

## Recommended Resources

- Introductions to open science and replication: Simmons et al. (2011), Vazire (2016), Chambers (2017); Engber (2017) Mellor et al. (2018), and Simmons et al. (2018).
- Guides on implementing open science practices: Hardwicke (2016), Crüwell et al. (2018), and Klein et al. (2018).
- Data on the consequences of teaching the replication crisis to undergraduates: Chopik et al. (2018).
- Podcasts: The Black Goat, ReproducibiliTea, EverythingHertz.

## Author Contributions

All authors listed have made a substantial, direct and intellectual contribution to the work, and approved it for publication.

### Conflict of Interest Statement

The authors declare that the research was conducted in the absence of any commercial or financial relationships that could be construed as a potential conflict of interest.

## References

[B1] Appelbaum M. Cooper H. Kline R. B. Mayo-Wilson E. Nezu A. M. Rao S. M. (2018). Journal article reporting standards for quantitative research in psychology: The APA publications and communications board task force report. Am. Psychol. 73, 3–25. 10.1037/amp0000191 29345484

[B2] Chambers C. D. (2017). The Seven Deadly Sins of Psychology: A Manifesto for Reforming the Culture of Scientific Practice. Princeton, NJ: Princeton University Press.

[B3] Chopik W. J. Bremner R. H. Defever A. M. Keller V. N. (2018). How (and Whether) to teach undergraduates about the replication crisis in psychological science. Teach. Psychol. 45, 158–163. 10.1177/0098628318762900

[B4] Crüwell S. van Doorn J. Etz A. Makel M. C. Moshontz H. Niebaum J. C. (2018). 8 Easy steps to open science: An annotated reading list. PsyArXiv [Preprint]. 10.31234/osf.io/cfzyx

[B5] DeHaven A. (2017). Preregistration: A Plan, Not a Prison. Available online at: https://cos.io/blog/preregistration-plan-not-prison/ (Accessed Nov 1, 2018)

[B6] Engber D. (2017). Daryl Bem Proved ESP Is Real. Which Means Science Is Broken. Available online at: https://slate.com/health-and-science/2017/06/daryl-bem-proved-esp-is-real-showed-science-is-broken.html (Accessed November 20, 2018).

[B7] Hardwicke T. (2016). A Pre-Registration Primer. Available online at: https://osf.io/8uz2g/ (Accessed November 1, 2018).

[B8] Houtkoop B. L. Chambers C. Macleod M. Bishop D. V. M. Nichols T. E. Wagenmakers E.-J. (2018). Data sharing in psychology: a survey on barriers and preconditions. Adv. Methods Pract. Psychol. Sci. 1, 70–85. 10.1177/2515245917751886

[B9] John L. K. Loewenstein G. Prelec D. (2012). Measuring the prevalence of questionable research practices with incentives for truth telling. Psychol. Sci. 23, 524–532. 10.1177/0956797611430953 22508865

[B10] Kerr N. L. (1998). HARKing: hypothesizing after the results are known. Personal. Social Psychol. Rev. 2, 196–217. 15647155 10.1207/s15327957pspr0203_4

[B11] Kidwell M. C. Lazarević L. B. Baranski E. Hardwicke T. E. Piechowski S. Falkenberg L.-S.. (2016). Badges to acknowledge open practices: a simple, low-cost, effective method for increasing transparency. PLoS Biol. 14:e1002456. 10.1371/journal.pbio.1002456 27171007 PMC4865119

[B12] Klein O. Hardwicke T. E. Aust F. Breuer J. Danielsson H. Hofelich Mohr A. (2018). A practical guide for transparency in psychological science. Collab. Psychol. 4:20 10.1525/collabra.158

[B13] Lindsay D. S. Simons D. J. Lilienfeld S. O. (2016). Research Preregistration 101. APS Observer, 29. Accessed online at: https://www.psychologicalscience.org/observer/research-preregistration-101#.WNuVNhLyuLI

[B14] McKiernan E. C. Bourne P. E. Brown C. T. Buck S. Kenall A. Lin J.. (2016). How open science helps researchers succeed. eLife 5, 1–19. 10.7554/eLife.16800 27387362 PMC4973366

[B15] Mellor D. T. Vazire S. Lindsay D. S. (2018). Transparent science: a more credible, reproducible, and publishable way to do science. PsyArXiv [Preprint]. 10.31234/osf.io/7wkdn

[B16] Nosek B. A. Ebersole C. R. DeHaven A. C. Mellor D. T. (2018). The preregistration revolution. Proc. Natl. Acad. Sci. U. S. A 115, 2600–2606. 10.1073/pnas.1708274114 29531091 PMC5856500

[B17] Open Science Collaboration (2015). Estimating the reproducibility of psychological science. Science 349:aac4716 10.1126/science.aac4716 26315443

[B18] R Core Team (2016). R: A Language and Environment for Statistical Computing. R Foundation for Statistical Computing. Available online at: http://www.R-project.org/

[B19] Rosenthal R. (1979). The file drawer problem and tolerance for null results. Psychol. Bull. 86, 638–641. 10.1037//0033-2909.86.3.638

[B20] Simmons J. P. Nelson L. D. Simonsohn U. (2011). False-positive psychology: undisclosed flexibility in data collection and analysis allows presenting anything as significant. Psychol. Sci. 22, 1359–1366. 10.1177/0956797611417632 22006061

[B21] Simmons J. P. Nelson L. D. Simonsohn U. (2018). False-Positive Citations. Perspec. Psychol. Sci. 13, 255–259. 10.1177/1745691617698146 29592640

[B22] Vazire S. (2016). Society for the Improvement of Psychological Science. Inflection Point with Schiller, Lauren. Available online at: https://www.inflectionpointradio.org/episodes/2016/8/3/simine-vazire-associate-professor-of-psychology-uc-davis

[B23] Vazire S. (2018). Implications of the credibility revolution for productivity, creativity, and progress. Persp. Psychol. Sci. 13, 411–417. 10.1177/1745691617751884 29961410

[B24] Wagenmakers E.-J. Wetzels R. Borsboom D. van der Maas H. L. J. Kievit R. A. (2012). An agenda for purely confirmatory research. Perspec. Psychol. Sci. 7, 632–638. 10.1177/1745691612463078 26168122

